# Predictors of Postoperative Hyponatremia in Patients Undergoing Head and Neck Surgery

**DOI:** 10.1007/s13193-025-02437-y

**Published:** 2025-09-30

**Authors:** Latika Kansal, Natarajan Ramalingam, Satadru Roy, Deepa Nair, Pankaj Chaturvedi, Vidisha Tuljapurkar

**Affiliations:** 1 Department of Head Neck Surgical Oncology, Tata Memorial Centre, Mumbai, India; 2 Department of Head and Neck Surgical Oncology, Christian Medical College, Vellore, India; 3 Department of Head and Neck Surgical Oncology, Manipal Hospital, Rangapani, Siliguri, India; 4 Department of Head and Neck Surgical Oncology, ACTREC, Navi Mumbai, India

**Keywords:** Hyponatremia, Head and neck, Postoperative, Predictors

## Abstract

Hyponatremia (HN) is a frequent postoperative occurrence after different surgeries, with potential impacts on recovery, hospital stay, and morbidity. Although frequently encountered, the literature regarding hyponatremia in head and neck cancer (HNC) patients remains scarce. The present study is conducted to evaluate the incidence, severity, and predictors of postoperative hyponatremia in HNC patients. A retrospective cohort study was conducted on 1058 patients who underwent head and neck surgeries between January 2019 and December 2020 at a tertiary care cancer center. Demographic, clinical, surgical, and biochemical data were analyzed from electronic medical records (EMR). Hyponatremia was classified as mild (130–135 mEq/L), moderate (125–130 mEq/L), or severe (< 125 mEq/L). Univariate and multivariate analyses were used to identify the predictors of HN. Classification and Regression Tree (CART) models were developed to form an algorithm for clinical decision making. Postoperative HN was seen in 67% of patients, with 32% classified as moderate-to-severe HN. Surgical reconstruction (OR 2.15, *p* < 0.001), intraoperative blood loss > 475 mL (OR 1.54, *p* = 0.01), fluid replacement > 1650 mL (OR 1.51, *p* = 0.014), and Charlson comorbidity index > 3 (OR 1.51, *p* = 0.016) were significant predictors of HN. Syndrome of inappropriate antidiuretic hormone secretion (SIADH) was identified in 9.3% of cases. CART analysis accurately predicted postoperative hyponatremia (69.5%) and SIADH (86%) based on perioperative variables. Hyponatremia is a frequent and potentially serious complication following head and neck cancer surgery. Surgical complexity, comorbidities, and intraoperative fluid shifts are key risk factors. Early recognition and appropriate perioperative management can help mitigate associated complications.

**Supplementary Information:**

The online version contains supplementary material available at 10.1007/s13193-025-02437-y.

## Background

Head and neck squamous carcinoma (HNSCC) accounts for approximately one-third of the cancer burden in India. Most of these cancers, especially the advanced cases, would undergo surgical resection. Major head and neck resections and reconstructions are prolonged surgeries and are associated with significant surgical complications and nonsurgical complications. Along with pulmonary and infective complications, electrolyte disturbances are commonly encountered in the postoperative period. With the reported incidence of 25–45% [1, 2] hyponatremia is associated with a longer hospital stay, perioperative complications among adults undergoing major surgery of any type, and an increased risk of 30-day mortality [2, 3]. Various pathophysiological explanations have been put forward for hyponatremia in patients with HNSCC [4]. Although frequently encountered, the literature regarding the diagnosis and management of hyponatremia in head and neck cancer patients remains scarce. 

The aim of this study is to look at the incidence and predictors of postoperative hyponatremia in patients undergoing head and neck surgery and to review the management guidelines for the same.

## Materials and Methods

A retrospective chart review of a prospectively maintained surgical database in a single head-and-neck surgical unit from a tertiary cancer center between January 1, 2019, and December 31, 2020, was performed.

Demographic details like age, gender, tumor site, stage, comorbidities, history of tobacco or alcohol consumption, body mass index, etc., were recorded. Details of the surgery like the duration of surgery, intraoperative blood loss, fluid replacement, postoperative serum sodium levels, length of hospital stay, complications, and management of hyponatremia were retrieved from the Electronic Medical Records of our hospital (EMR).

The definitions of hyponatremia were considered in accordance with the cut-offs provided by Spasovski G et al. Hyponatraemia Guideline Development Group. [5]

Normonatremia was defined as serum sodium levels between 135 and 145 mEq/L or 135 to 145 mmol/L. Mild Hyponatremia was defined as levels between 130 and 135 mEq/L. Moderate Hyponatremia was defined as levels between 125 and 130 mEq/L, while severe hyponatremia was defined as values < 125 mEq/L. The definition of SIADH was taken in accordance with Schwartz-Bartter Clinical criteria [6].

1. Decreased measured serum osmolality (< 275 mOsm/kg H2O)
2. Clinical euvolemia
3. Urinary osmolality > 100 mOsm/kg H2O
4. Urinary [Na +] > 40 mmol/L with normal dietary sodium intake

As per the institutional protocols, patients undergoing surgery for cancers of the oral cavity, laryngopharynx, and oropharynx were started on nasogastric feeding on the first postoperative day. Patients with surgeries not involving the aerodigestive tract were started on oral diet on the same or next postoperative day.

Serum electrolyte values were checked on the first/second postoperative day and every 3–4 days thereafter as deemed necessary. Patients detected to have hyponatremia were started on salt supplementation in their feeds and appropriate replacement with intravenous fluids as per needs.

## Statistical Analysis

Statistical analysis was performed using IBM SPSS© software version 24.0, and the results were expressed in frequency and percentages as appropriate. Overall, the incidence of hyponatremia in the entire cohort and that in patients with oral or nasogastric feeding was calculated, and the degree of hyponatremia, i.e., mild, moderate, or severe, was recorded.

We excluded the patients with incomplete data for demographic information, intraoperative details, and postoperative values of serum sodium and management.

Univariate analysis (UVA) and logistic regression analysis were performed to analyze the predictive factors for hyponatremia and SIADH. Receiver operating curves (ROC) and area under the curve (AUC) were generated to predict fluid replacement volumes and blood loss, which were significant for developing postoperative hyponatremia. The Classification and Regression Tree (CART) analysis model was created using R software to create decision trees and predictions for postoperative hyponatremia and SIADH using variables that were found to be significant on univariate and logistic regression analysis. CART is a non-parametric decision tree algorithm introduced by Breiman et al. in 1986 [7]. It recursively partitions the dataset into subsets based on predictor variables, creating a tree structure where each terminal node (leaf) represents a predicted outcome. The tree structure provides a clear and understandable visualization of decision-making processes.

## Results

### Demographics

The study included 1058 patients with a median age of 48 years (range 14–80 years) A large number of patients (62%) had a Charlson comorbidity index score > 3, indicating associated comorbidities, and 27% of patients receiving prior treatment in the form of surgery, chemotherapy, and radiation. In the entire cohort (*n* = 1058), 30% had hyponatremia in the preoperative period. The demographic details of patients are summarized in Table 1.

**Table 1:** Demographic details

Parameter	Subsets	*N* = 1058 (%)
Age	< 60 yrs More than 60 years	860 (81.3%) 198 (18.7%)
Sex	Female Male	250 (23.6%) 808 (76.4%)
BMI^a^[8]	Normal 18.5–24.9 kg/m^2^ Underweight < 18.5 kg/m^2^ Overweight/obesity > 24.9 kg/m^2^	560 (52.9%) 114 (10.8%) 384 (36.3%)
Comorbidities	Diabetes mellitus Hypertension DM + hypertension Heart disease Pulmonary disease Hepatitis Hypothyroidism	55 (5.2%) 115 (10.9%) 51 (4.8%) 12 (1.1%) 13 (1.2%) 3 (0.3%) 35 (3.3%)
Charlson comorbidity index	< 3 More than 3	404 (38.2%) 654 (61.8%)
Alcohol and tobacco use	No Yes	299 (28.3%) 759 (71.7%)
Stage of tumor	Early (stages I and II) Advanced (stages III and IV)	539 (50.9%) 519 (49.1%)
Prior treatment	None Surgery Non-Surgery (RT/CTRT and NACT^b^)	770 (72.8%) 179 (16.9%) 109 (10.3%)
Reconstruction	None Pedicled flap/local flap Free flap	517 (48.9%) 331 (31.3%) 210 (19.8%)
Type of postoperative feeds	Tube feeds Oral feeds	841 (79.5%) 217 (20.5%)
Pre operative hyponatremia	Yes Mild Moderate Severe	315 (29.8%) 198 (62.8%) 117 (37.1%) 0

^a^*BMI* body mass index, ^b^*NACT* neoadjuvant chemotherapy

### Incidence of Hyponatremia

The overall incidence of hyponatremia (HN) was 67% (708 /1058 patients) and the majority of these patients 68% (479/708) had mild hyponatremia defined as serum sodium levels between 130 and 135 mEq/L.

However, 22% (155/708) and 10% (74/708) patients developed moderate and severe hyponatremia, respectively, that required detailed monitoring and active management for correction of hyponatremia. Incidence of HN was significantly higher (72%) in the patients that received nasogastric tube (NGT) feeding.

A large percentage (68%) of patients had mild hyponatremia that was a transient biochemical anomaly detected on routine examination and was clinically asymptomatic.

However, about one third of the patients (32%) developed moderate to severe postoperative HN that required more aggressive monitoring and medical management. Hence, we looked at predictive factors to identify the patients at risk of developing significant HN.

Among the patients that developed postoperative moderate to severe HN, 99 (14%) cases developed SIADH and were managed with fluid restriction (100%), hypertonic (3%) saline infusions (28.2%), and selective, competitive vasopressin receptor 2 antagonist—Tolvaptan (43.4%) depending on the severity of hyponatremia and the response to treatment.

### Factors Associated with the Development of Hyponatremia

**Fig. 1:**
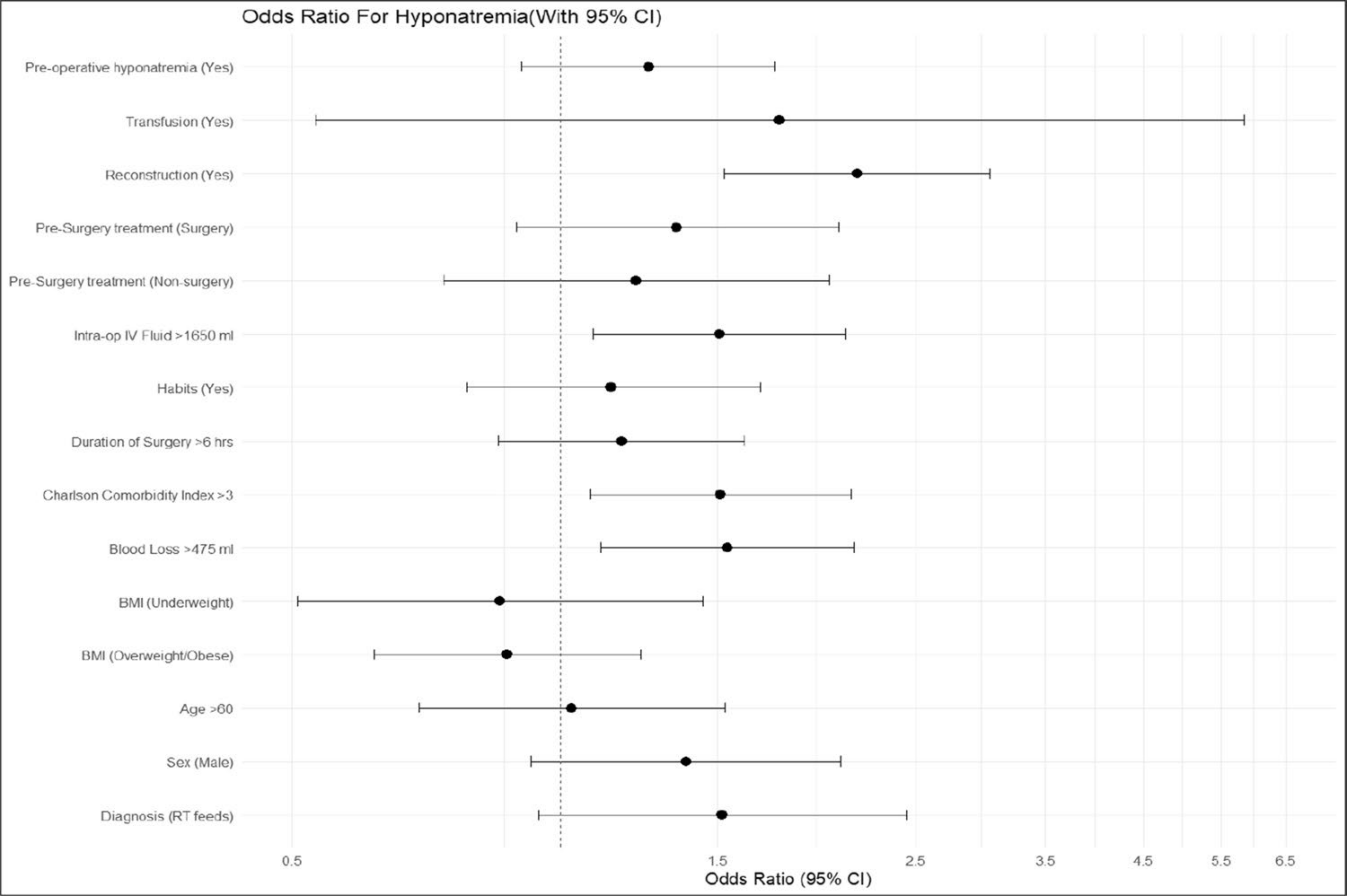
Forest plot chart with Odds ratio for variables predicting postoperative hyponatremia

Receiver operating curves (ROC) generated (Supplementary_Fig.1). To identify intraoperative factors predisposing for development of HN indicated that surgeries with higher blood loss (> 475 mL), large volumes of intraoperative fluid replacement (> 1650 mL) and longer duration (> 6 h) were at a higher risk of developing significant postoperative HN, which was confirmed on logistic regression analysis (Fig. 1).

As a large majority of the cases that developed postoperative HN were mild (130-135 mEq/L), asymptomatic, and transient, we identify the factors predicting the development of significant (moderate/severe) HN. In the entire cohort, on univariate analysis, the presence of preoperative hyponatremia, nasogastric feeding, Charlson index > 3, addiction, prolonged surgeries > 6 h, blood loss > 475 mL, or large intravenous fluid volume were shown to have a higher risk of developing hyponatremia postoperatively (Supplementary_Table.1). However, in MVA, only preop HN and recon were seen as predictors of significant HN (supplementary Table 2).

We performed logistic regression and CART analysis on the cohort that had any grade of HN (*n* = 708) to identify the factors that predicted the development of moderate/severe HN. On multivariate logistic regression analysis, patients with multiple comorbidities (OR − 1.51), higher blood loss (OR − 1.53), and large intraoperative volume replacements (OR − 1.50)) were at a higher risk of developing HN, whereas patients undergoing surgeries with flap reconstructions were at the highest risk (OR − 2.0) (Table 2) (Fig. 1).

**Table 2:** Logistic regression for predictors of hyponatremia

Variable		Total (708)	Event (229)	OR (95%CI)	*P* value
Preoperative hyponatremia	No Yes	458 250	140 89		0.172
				1.255 (0.904 1.738)	
Reconstruction	No reconstruction	271	61		
	Reconstruction	437	168	2.150 (1.524, 3.033)	< *0.001*
Intraoperative fluid replacement	< 1650 mL	291	79		
	> 1650 mL	417	150	1.508 (1.087, 2.090)	*0.014*
Alcohol and tobacco use	No Yes	163 545	49 180	1.14 (0.785, 1.677)	0.478
Duration of surgery	< 6 h > 6 h	408 300	126 103	1.17 (0.852, 1.608)	
					0.332
Charlson comorbidity index	< 3 > 3	258 450	69 160	1.511 (1.079, 2.116)	
					*0.016*
Blood loss	< 475 mL > 475 mL	290 418	78 151	1.537 (1.108, 2.133)	
					*0.01*
Sex	Female Male	152 556	41 188	1.383 (0.928, 2.061)	
					0.11
Type of feed	Oral feeds RT feeds	104 604	26 203	1.519 (0.945, 2.442)	
					0.085

### Diagnosis and Management—CART Algorithm

**Fig. 2:**
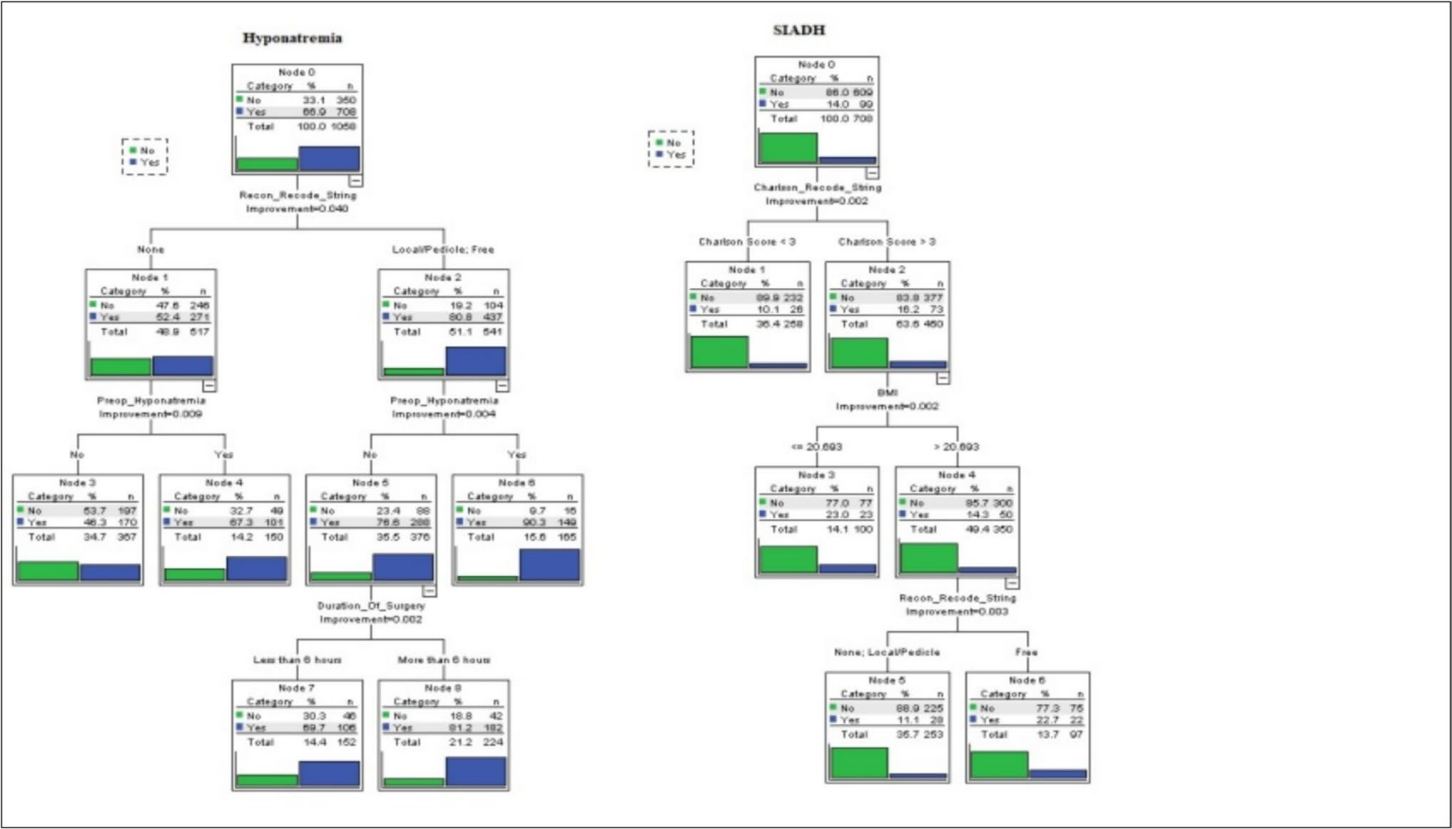
CART algorithm for prediction of hyponatremia

The Classification and Regression Tree (CART) algorithm was used to create decision trees and predictions for Hyponatremia (Fig. 2). The most important predictor variables for Hyponatremia using the CART method included reconstruction, duration of surgery, and preoperative sodium levels in descending order.

Patients undergoing reconstructive procedures had a significantly greater incidence of postoperative hyponatremia, irrespective of the type of reconstruction performed (*p* < 0.040).

The duration of surgery > 6 h was found to have a significant impact on postoperative hyponatremia and preoperative hyponatremia (*p* < 0.013). Patients have more chances of significant postoperative hyponatremia shown in the right decision tree (Fig. 2). However, patients with a Charlson comorbidity index > 3, BMI < 20 kg/m2, and patients who underwent reconstruction have significant chances for SIADH shown in the left decision tree (Fig. 2). The accuracy of CART for predicting hyponatremia and SIADH was 69.5% and 86%, respectively.

Each decision tree is a flowchart-like structure where Nodes represent variables found significant on logistic regression, green bars show absence of hyponatremia, and blue bars represent the presence of hyponatremia.

## Discussion

Hyponatremia is one of the most common electrolyte disturbances encountered in the medical and surgical patient population. Overall incidence is as high as 15%–30% [9], mainly seen in geriatric patients (7%−53%) [9], those with chronic renal and liver diseases (up to 50%) [10]. In surgical patients, although it can occur after any major surgery, the majority of the available literature is from orthopedic, cardiac, and GI surgeries. Studies regarding hyponatremia in oncologic surgeries, especially head and neck cancers, are sparse.

The reported incidence of postoperative HN ranges from 18 to 30% [1, 11] in the current literature. In our study, clinically significant HN (< 130 mEq) was seen in 32% of patients. Although mild asymptomatic HN is far more common, symptoms of hyponatremia may be confused with normal postoperative reactions such as nausea, headache, gait instability, attention impairment, and falls [12, 13]. Cerebral edema resulting in disturbances in consciousness, including drowsiness or even delirium, is a major complication resulting from severe HN postoperatively [14]. Hyponatremia also has been associated with increased perioperative complications like pneumonia, wound infections, and cardiac events, as well as prolonged hospitalization [3].

Therefore, identifying early predictors of postoperative hyponatremia in patients undergoing head and neck surgery is crucial to prevent and manage this condition effectively.

### Pathophysiology of Postoperative Hyponatremia

The diagnosis of the etiology of hyponatremia is difficult because multiple mechanisms of hyponatremia may be occurring simultaneously in the postoperative period. Postoperative hypovolemia results in hyponatremia due to blood loss, inadequate replacement, and further surgical stress itself results in the release of the vasopressin secretion, which acts on the kidney, leading to water retention to compensate for hypovolemia, resulting in dyselectrolytemia [15]. Studies suggest that squamous cell carcinomas enhance the production of neuropeptide Y [16] along with the use of anesthetic drugs, pain, pyrexia state, and hypovolaemic condition, which can further elevate the release of antidiuretic hormone (ADH) [11].

Intraoperative fluid overload in patients with already enhanced baseline ADH levels further exacerbates the postoperative hyponatremia [17]. Transfusion with a large volume of fluids leads to dilutional hyponatremia [18], and stored blood products can also change electrolyte concentrations, potentially leading to hyponatremia after transfusion [19]. The need for blood transfusion is determined by the amount of blood loss, the hemoglobin concentration, and the patient's clinical condition [20]. Blood transfusion, along with enhanced fluid resuscitation during the surgical procedures, leads to a state of enhanced fluid surplus This, in turn, leads to a similar cascade of events that precipitate hyponatremia in these patients, along with prolonged water retention for several days [21], which ultimately exacerbates the hyponatremic state. Furthermore, head and neck patients on tube feeding may have more hypoalbuminemia triggers secretion of ADH, making patients more prone to hyponatremia [22]. A higher incidence of hyponatremia is reported with more invasive surgeries, preoperative hyponatremia, preoperative undernutrition, old age, and low BMI [23, 24]. In our study, similar findings were seen, where intraoperative factors like prolonged surgeries, as seen in major reconstructive procedures, preoperative hyponatremia, and comorbidities were associated with significant risk of postoperative hyponatremia.

### ADH and Hyponatremia

As discussed previously, postoperative rise in ADH is multifactorial. Up to 4.4% of surgical patients have hyponatraemia [25]; however, the prevalence of SIADH is reported as 7–16% in various malignancies. Talmi et al. noted a 3% incidence of SIADH in 1436 squamous cell carcinoma head and neck patients [26]. Mesko et al. reported in a prospective study that SIADH develops more in patients undergoing neck dissection with jugular vein ligation, preoperative radiation therapy, and squamous cell cancer [11]. Our study shows SIADH incidence of 9.3% in the entire cohort.

## Resuscitation Fluids and Transfusion Related to Hyponatremia

Ziegler reported 4.6% metabolic complication with every 500 mL increase in intravenous fluid administration [27]. Similarly, our study shows patients undergoing blood transfusions due to blood loss > 475 mL (*p* = 0.011) and enhanced intravenous transfusions > 1650 mL (*p* = 0.014) had a significantly higher incidence of hyponatraemic state postoperatively.

On CAR T analysis as well, patients undergoing prolonged surgeries > 6 h (*p* = 0.002) including reconstructive procedures (*p* < 0.040) had a higher incidence of both postoperative hyponatremia and SIADH in our study, as it involves a higher IV fluid infusion, which might explain the increased risk. Low BMI patients < 20 kg/m^2^ (*p* = 0.002) and patients with a Charlson comorbidity index score > 3 had significantly more chances of having postoperative SIADH.

### Hyponatremia and the Inpatient Stay

Development of postoperative HN can also result in prolonged inpatient stay, ICU admission for correction of electrolyte imbalances, with its implications for resource utilization, treatment costs, and delay in the adjuvant treatment planning.

As reported in various studies, in hyponatremic patients average length of stay is between 1.44 and 9.2 days and the mortality rate is between 2.1 and 28.1% in various studies [28, 29]. Upon admission, if sodium levels equal to or lower than 130 mEq/l are associated with a poor prognosis in surgical outcomes of the patient [1, 12].

In our study, the duration of hospital stay was also prolonged in patients with hyponatremia (9.6 days vs 11.36 days). Metanalysis by Teo et al. reported an odds ratio of 1.37 with preoperative hyponatremia and increased duration of hospital stay [30]. Similar findings by Deitelzweig et al. indicate that hyponatremia is associated with increased length of stay, 30-day readmission rates, and hospital cost [31].

In the present cohort, all of 26 (3.5%) cases that required readmission to the ICU for management of HN had been diagnosed with SIADH, and the majority (78%) had developed severe hyponatremia (Na- < 125 m Eq/L) thus requiring prolongation of hospital stay.

There are mixed reports regarding the risk of death due to HN in the postoperative period, where some studies have reported up to 7% incidence of postoperative mortality [23, 24]; other studies have found no association. In our study, there was one death due to severe hyponatremia unresponsive to treatment.

### Significance of Preoperative HN

In our study, the presence of preoperative hyponatremia had a significantly greater chance of postoperative low serum sodium levels (*p* = 0.04). Among these preoperative HN was Mild (62.9%), Moderate (37.1%) and Severe (0%) in these patients. Preexisting hyponatremia due to difficulty in swallowing, preoperative tube feeding, and tumor-related inflammation [32] can exacerbate the postoperative chances of hyponatremia. Approximately 1 in every 13 patients planned for surgery has been reported to have preoperative hyponatremia [3]. It may reflect the presence of critical physiologic derangements such as sodium concentrations, which play an important role in maintaining electrical gradients across cell membranes or can affect cerebral function. A systematic review by Teo et al. suggested preoperative hyponatremia was 88% specific and 25% sensitive in predicting major complications, with a 2.5-fold increased risk of major complications after surgical procedures [30].

### Diagnosis and Management of Postoperative HN

As seen in the present study cohort, the majority of patients having postoperative HN develop mild HN (sr. Sodium 130–135 mEq/L) that is often transient and asymptomatic. These patients can be managed with the addition of extra salt in the diet and/or free water restriction through NGT. Patients with moderate to severe hyponatremia, however, need to be monitored more aggressively and possibly be investigated for SIADH, as they can develop serious complications like altered sensorium, cerebral edema, and convulsions, and require prolonged hospitalization.

**Fig. 3:**
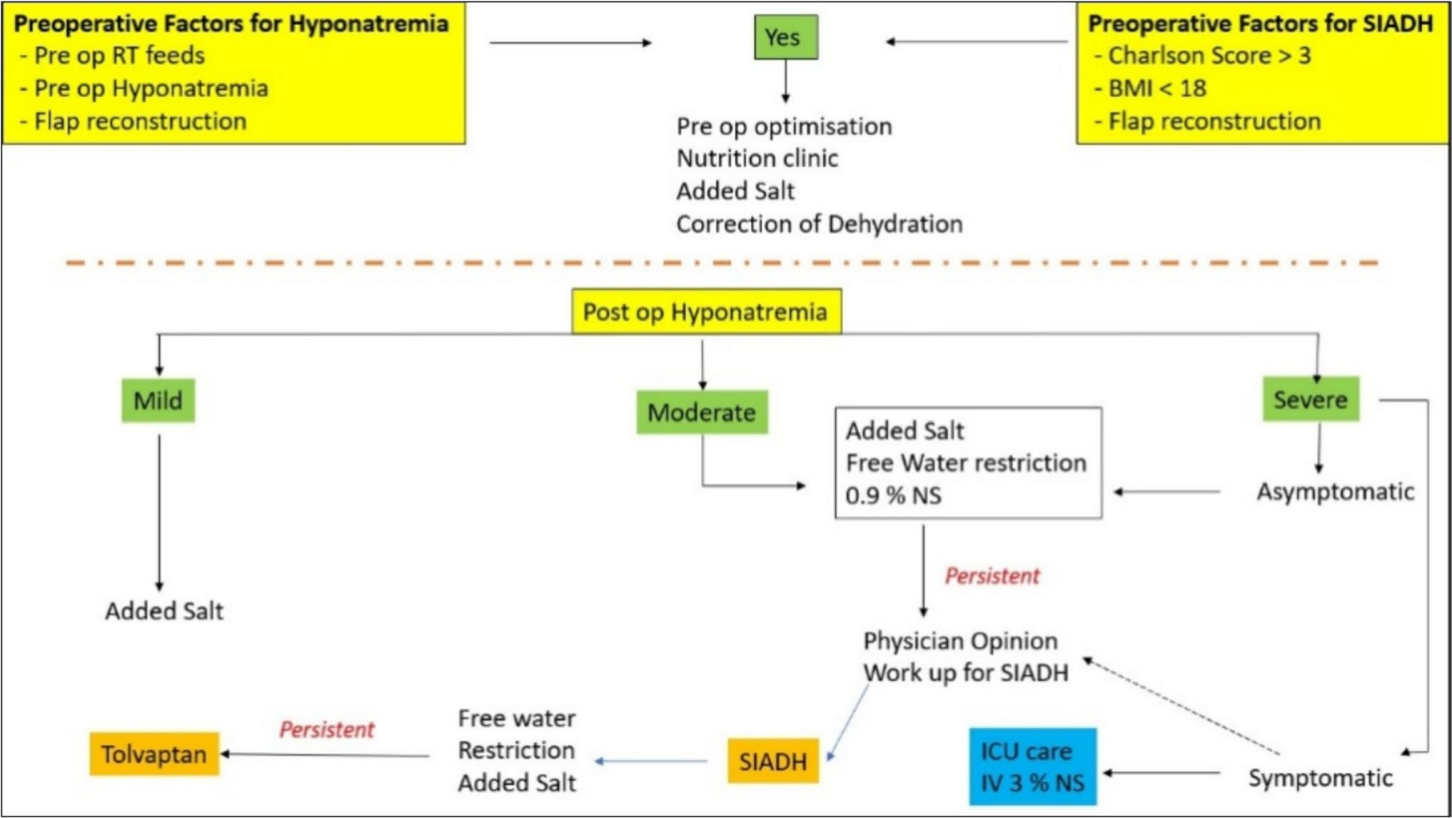
Algorithm for management of Hyponatremia and to detect patients at a higher risk of developing postoperative hyponatremia

Based on our experience, we suggest the following algorithm for management of HN to detect patients at a higher risk of developing postoperative HN and manage them effectively to minimize the severe complications and/or avoid ICU admissions for these patients (Fig. 3).

Strength of the present study is the large case cohort, with uniform treatment protocols in a single surgical unit, and robust statistical analysis. However, we have included all the subsites in the head and neck cancers, including both recurrent and treatment naïve cases. We also lacked details of medications that may possibly interfere with the development of hyponatremia. However, to the best of our knowledge, this is the largest study so far documenting the incidence and management of HN in head and neck cancer patients.

To summarize, postoperative HN is a common metabolic complication following surgery in head and neck cancer patients. Although the majority of these cases are mild and clinically asymptomatic, it is important to preoperatively identify the patients that are at a higher risk of HN. Early diagnosis and management of postoperative HN is essential to prevent severe HN with its potentially life-threatening complications and treatment delays. 

## Supplementary Information

Below is the link to the electronic supplementary material.

ESM 1 (DOCX 70.9 KB)

ESM 2 (DOCX 22.4 KB)

## References

[1] Feinstein AJ, Davis J, Gonzalez L, Blackwell KE, Abemayor E, Mendelsohn AH (2016) Hyponatremia and perioperative complications in patients with head and neck squamous cell carcinoma. Head Neck 38(Suppl 1):E1370–E1374. 10.1002/hed.24242 26382762 10.1002/hed.24229

[2] Doshi SM, Shah P, Lei X, Lahoti A, Salahudeen AK (2012) Hyponatremia in hospitalized cancer patients and its impact on clinical outcomes. Am J Kidney Dis 59(2):222–228. 10.1053/j.ajkd.2011.10.041 22001181 10.1053/j.ajkd.2011.08.029

[3] Leung AA, McAlister FA, Rogers SO, Pazo V, Wright A, Bates DW (2012) Preoperative hyponatremia and perioperative complications. Arch Intern Med 172(19):1474–1481. 10.1001/archinternmed.2012.3992 22965221 10.1001/archinternmed.2012.3992

[4] Toro C, Rinaldo A, Silver CE, Politi M, Ferlito A (2010) Paraneoplastic syndromes in patients with oral cancer. Oral Oncol 46(1):14–18. 10.1016/j.oraloncology.2009.11.003 19932048 10.1016/j.oraloncology.2009.09.007

[5] Spasovski G, Vanholder R, Allolio B, Annane D, Ball S, Bichet D et al (2014) Clinical practice guideline on diagnosis and treatment of hyponatraemia. Eur J Endocrinol 170(3):G1–G47. 10.1530/EJE-13-1020 24569125 10.1530/EJE-13-1020

[6] Bartter FC, Schwartz WB (1967) The syndrome of inappropriate secretion of antidiuretic hormone. Am J Med 42(5):790–806. 10.1016/0002-9343(67)90065-2 5337379 10.1016/0002-9343(67)90096-4

[7] Breiman L, Friedman JH, Olshen RA, Stone CJ (1984) Classification and regression trees. Wadsworth10.1201/9781315139470

[8] Tan KCB (2004) Appropriate body-mass index for Asian populations and its implications for policy and intervention strategies. Lancet 363(9403):157–163. 10.1016/S0140-6736(03)15268-3 14726171 10.1016/S0140-6736(03)15268-3

[9] DeVita MV, Gardenswartz MH, Konecky A, Zabetakis PM (1990) Incidence and etiology of hyponatremia in an intensive care unit. Clin Nephrol 34(4):163–166 (**PMID: 2257702**) 2257702

[10] Angeli P, Wong F, Watson H, Ginès P (2006) Hyponatremia in cirrhosis: results of a patient population survey. Hepatology 44(6):1535–1542. 10.1002/hep.21412 17133458 10.1002/hep.21412

[11] Mesko TW, Garcia O, Yee LD, Villar M, Chan H (1997) The syndrome of inappropriate secretion of antidiuretic hormone (SIADH) as a consequence of neck dissection. J Laryngol Otol 111(5):449–453. 10.1017/S0022215100137363 9205607 10.1017/s0022215100137612

[12] Waikar SS, Mount DB, Curhan GC (2009) Mortality after hospitalization with mild, moderate, and severe hyponatremia. Am J Med 122(9):857–865. 10.1016/j.amjmed.2009.01.027 19699382 10.1016/j.amjmed.2009.01.027 PMC3033702

[13] Tambe AA, Hill R, Livesley PJ (2003) Post-operative hyponatraemia in orthopaedic injury. Injury 34(4):253–255. 10.1016/S0020-1383(02)00256-5 12667775 10.1016/s0020-1383(02)00256-5

[14] Gill G, Huda B, Boyd A, Skagen K, Wile D, Watson I et al (2006) Characteristics and mortality of severe hyponatraemia – a hospital-based study. Clin Endocrinol 65(2):246–249. 10.1111/j.1365-2265.2006.02583.x 10.1111/j.1365-2265.2006.02583.x 16886968

[15] Verbalis JG, Goldsmith SR, Greenberg A, Korzelius C, Schrier RW, Sterns RH et al (2013) Diagnosis, evaluation, and treatment of hyponatremia: expert panel recommendations. Am J Med 126(10):S1–S42. 10.1016/j.amjmed.2013.07.006 24074529 10.1016/j.amjmed.2013.07.006

[16] Lee JH, Cha MJ, Choi SH, Hwang SJ, Kim DG, Jahng JW (2004) Neuropeptide Y immunoreactivity and corticotropin-releasing hormone mRNA level are increased in the hypothalamus of mouse bearing a human oral squamous cell carcinoma. Neuropeptides 38(6):345–350. 10.1016/j.npep.2004.07.002 15567470 10.1016/j.npep.2004.07.004

[17] Shin CH, Long DR, McLean D, Grabitz SD, Ladha K, Timm FP et al (2018) Effects of intraoperative fluid management on postoperative outcomes: a hospital registry study. Ann Surg 267(6):1084–1092. 10.1097/SLA.0000000000002238 28288059 10.1097/SLA.0000000000002220

[18] Solhpour A, Kumar S, Koch MJ, Doré S (2023) Impact of blood component transfusions, tranexamic acid and fluids on subarachnoid hemorrhage outcomes. Brain Hemorrhages 4(2):77–95. 10.1016/j.hest.2022.07.002

[19] Clement OO, Safo ABK, Dogbe EE (2015) Changes in potassium and sodium concentrations in stored blood. Pan Afr Med J 20 10.11604/pamj.2015.20.236.585. 10.11604/pamj.2015.20.236.5851 PMC4919675 27386032

[20] Liumbruno, G. M., Bennardello, F., Lattanzio, A., Piccoli, P., Rossetti, G., & Italian Society of Transfusion Medicine and Immunohaematology Working Party (2011) Recommendations for the transfusion management of patients in the peri-operative period. III The post-operative period Blood Transfusion 9(3):320–335. 10.2450/2010.0067-10 21627922 10.2450/2011.0076-10 PMC3136601

[21] Kumar S, Berl T (1998) Sodium. Lancet 352(9123):220–228.10.1016/S0140-6736(98)01037-7 10.1016/S0140-6736(97)12169-9 9683227

[22] Tanemoto M (2008). Effect of serum albumin on serum sodium: necessity to consider the Donnan effect. QJM: An Int J Med 101(10):827–828.10.1093/qjmed/hcn093. 10.1093/qjmed/hcn104 18725371

[23] Kinoshita Y, Tamai K, Oka M, Habibi H, Terai H, Hoshino M et al (2022) Prevalence, risk factors, and potential symptoms of hyponatremia after spinal surgery in elderly patients. Sci Rep 12:18622. 10.1038/s41598-022-23583-1 36329205 10.1038/s41598-022-23583-1 PMC9633822

[24] Sanada M, Tominaga H, Kawamura I, Tokumoto H, Ogura T, Taniguchi N (2024) Incidence and risk factors for hyponatremia in postoperative spinal surgery patients. Spine Surg Relat Res 8(3):267–271. 10.22603/ssrr.2023-0158 38868792 10.22603/ssrr.2023-0158 PMC11165490

[25] Chung HM, Kluge R, Schrier RW, Anderson RJ (1986) Postoperative hyponatremia: a prospective study. Arch Intern Med 146(2):333–336. 10.1001/archinte.146.2.333 3947194

[26] Talmi YP, Hoffman HT, McCabe BF (1992) Syndrome of inappropriate secretion of arginine vasopressin in patients with cancer of the head and neck. Ann Otol Rhinol Laryngol 101(11):946–949. 10.1177/000348949210101111 1332568 10.1177/000348949210101111

[27] Ziegler A, Carollo E, Adams W, Bier-Laning C (2023) The total amount of fluid administered is associated with postoperative complications in head and neck cancer surgery. World Journal of Otorhinolaryngology - Head and Neck Surgery 9(4):288–294. 10.1002/wjo2.86 38059145 10.1002/wjo2.86 PMC10696273

[28] Nair V, Niederman MS, Masani N, Fishbane S (2007) Hyponatremia in community-acquired pneumonia. Am J Nephrol 27(2):184–190. 10.1159/000100856 17356253 10.1159/000100866

[29] García Segura A, Gadea Ruiz C, Oliva Fanlo B, Ruiz Rodríguez R, Antón Botella F, Pinilla Moraza J et al (1994) Hyponatremia upon admission in patients over 65 years of age: Relation with medium length of stay and hospital mortality. An Med Interna 11(10):487–489 (**PMID: 7865655**) 7865655

[30] Teo CB, Gan MY, Tay RYK, Loh WJ, Loh NHW (2023) Association of preoperative hyponatremia with surgical outcomes: a systematic review and meta-analysis of 32 observational studies. J Clin Endocrinol Metab 108(5):1254–1271. 10.1210/clinem/dgad014 36472931 10.1210/clinem/dgac685 PMC10099166

[31] Deitelzweig S, Amin A, Christian R, Friend K, Lin J, Lowe TJ (2013) Health care utilization, costs, and readmission rates associated with hyponatremia. Hosp Pract 41(1):89–95. 10.3810/hp.2013.02.1014 10.3810/hp.2013.02.1014 23466971

[32] Kim JH, Park JH, Eisenhut M, Yu JW, Shin JI (2016) Inflammasome activation by cell volume regulation and inflammation-associated hyponatremia: a vicious cycle. Med Hypotheses 93:117–121. 10.1016/j.mehy.2016.05.003 27372869 10.1016/j.mehy.2016.05.018

