# Supplementary material for: Predictors of Postoperative Hyponatremia in Patients Undergoing Head and Neck Surgery
Source: Indian J Surg Oncol. 2025 Sep 30;17(6):1369–77. doi: 10.1007/s13193-025-02437-y (PMC13315074; doi:10.1007/s13193-025-02437-y)
Supplement: Supplementary file 1 — (DOCX 70.9 KB) [file 13193_2025_2437_MOESM1_ESM.docx]

**Supplementary figures for Predictors of postoperative Hyponatremia in patients undergoing head and neck surgery**

**Authors:** Dr Latika Kansal, Dr Natarajan Ramalingam, Dr Satadaru Roy, Dr Deepa Nair, Dr Pankaj Chaturvedi**.**

**Corresponding Author: Dr Vidisha Tuljapurkar,

**Professor in Head Neck Surgical oncology, Tata Memorial Centre, Mumbai, India. Email: [vidishavt@yahoo.com](mailto:vidishavt@yahoo.com)

**Indian Journal of Surgical oncology**

**Manuscript id: IJSO-D-25-00383**

Supplementary_Fig.1: Receiver operating curves for intraoperative fluid replacement, blood loss and duration of surgery respectively.


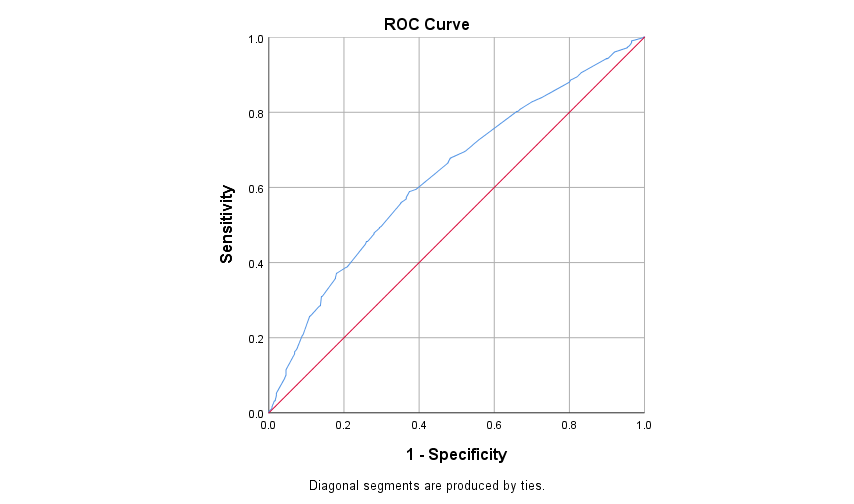

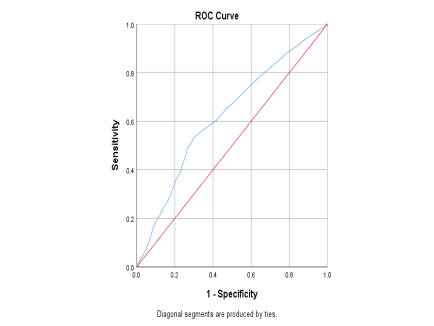

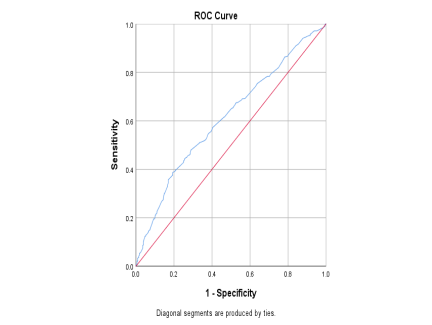


Blue curve represents the model's discriminative ability and red diagonal line denotes a no-discrimination line generated to find out cut of values for intraoperative volume replacement, blood loss and duration of surgery respectively.
