# Supplementary material for: Predictors of Postoperative Hyponatremia in Patients Undergoing Head and Neck Surgery
Source: Indian J Surg Oncol. 2025 Sep 30;17(6):1369–77. doi: 10.1007/s13193-025-02437-y (PMC13315074; doi:10.1007/s13193-025-02437-y)
Supplement: Supplementary file 2 — (DOCX 22.4 KB) [file 13193_2025_2437_MOESM2_ESM.docx]

**Supplementary tables for Predictors of postoperative Hyponatremia in patients undergoing head and neck surgery**

**Authors:** Dr Latika Kansal, Dr Natarajan Ramalingam, Dr Satadaru Roy, Dr Deepa Nair, Dr Pankaj Chaturvedi**.**

**Corresponding Author: Dr Vidisha Tuljapurkar,

**Professor in Head Neck Surgical oncology, Tata Memorial Centre, Mumbai, India. Email: [vidishavt@yahoo.com](mailto:vidishavt@yahoo.com)

**Indian Journal of Surgical oncology**

**Manuscript id: IJSO-D-25-00383**

Supplementary_Table.1: Univariate analysis of predictors for significant hyponatremia

|  | | Mild+  No Hyponatremia  (829) | Mod/Severe hyponatremia  (229) | p value |
| --- | --- | --- | --- | --- |
| Reconstruction | Yes  No | 373 (45%)  456 (55%) | 168 (73.4%)  61 (26.6%) | **<0.01** |
| Transfusion | Yes  No | 18(2.1%)  811(97.9%) | 5 (2.2%)  224 (97.8%) | 0.9 |
| Neck dissection | Yes  No | 676 (81.5%)  153 (18.5%) | 192 (83.8%)  37 (16.1%) | 0.42 |
| Blood loss | <= 475 ml  > 475 ml | 424 (51.1%)  405 (48.9%) | 78 (34.1%)  151 (65.9%) | **<0.01** |
| Duration of surgery | <=6hrs  > 6hrs | 550 (66.4%)  279 (33.6%) | 126 (55%)  103 (45%) | **0.002** |
| Intraoperative fluid replacement | < =1650 ml  > 1650 ml | 431 (52%)  398 (48%) | 79 (34.5%)  150 (65.5%) | **<0.01** |
| Alcohol and tobacco use | No  Yes | 250 (30.1%)  579 (69.9%) | 49 (21.4%)  180 (78.6%) | **0.009** |
| Age | <=60  >60 | 677(81.7%)  152 (18.3%) | 183 (80%)  46 (20%) | 0.54 |
| Type of feed | Oral feeds  RT feeds | 191 (23%)  638 (77%) | 26 (11.4%)  203 (88.6%) | **<0.01** |
| Charlson Co-Morbidity Index | <=3  > 3 | 335 (40.4%)  494 (59.6%) | 69 (30.1%)  160 (69.9%) | **0.005** |
| Pre-Surgery  treatment | No treatment  Surgery  Non-surgical treatment | 614 (74.1%)  134 (16.2%)  81 (9.7%) | 156 (68.2%)  45 (19.6%)  28 (12.2%) | 0.21 |
| BMI | Normal 18.5 kg/m^2^-24.9 kg/m^2^  Underweight <18.5 kg/m^2^  Overweight/Obesity>24.9 kg/m^2^ | 431 (52%)  90 (10.9%)  308 (37.1%) | 129 (56.3%)  24 (10.5%)  76 (33.2%) | 0.48 |
| Sex | Female  Male | 209 (25.2%)  620 (74.8%) | 41 (17.9%)  188 (82.1%) | 0.02 |
| Pre-operative hyponatremia | No  Yes | 603 (72.7%)  226 (27.3%) | 140 (61.1%)  89 (38.9%) | 0.001 |

Supplementary_Table 2. Multivariate analysis of Predictors of Significant hyponatremia

| **Variable (n=1058)** | **Level** | **OR(95% CI)** | **p.value** |
| --- | --- | --- | --- |
| PreopHyponatremia | No  Yes | 1.679(1.221,2.309) |  |
|  |  |  | **0.001** |
| Reconstruction | No  Yes | 3.104(2.035,4.735) |  |
|  |  |  | **<0.001** |
| IVF | < 1650 ml  > 1650 ml | 1.394(0.897,2.165) |  |
|  |  |  | 0.14 |
| Habits | None  Yes | 0.941(0.604,1.466) |  |
|  |  |  | 0.899 |
| Duration | < 6hrs  > 6hrs | 0.731(0.488,1.097) |  |
|  |  |  | 0.13 |
| Charlson | < 3  > 3 | 1.365(0.982,1.897) |  |
|  |  |  | 0.064 |
| Blood loss | < 475 ml  > 475 ml | 1.048(0.700,1.571) |  |
|  |  |  | 0.819 |
| Sex | Female  Male | 0.999(0.652,1.531) |  |
|  |  |  | 0.997 |
| Diagnosis | Oral feeds  RT feeds | 1.156(0.637,2.1) |  |
|  |  |  | 0.633 |
